# Primary tuberculosis of the tongue

**DOI:** 10.1590/0037-8682-0514-2021

**Published:** 2021-12-17

**Authors:** Allan Vinícius Martins-de-Barros, Emanuel Dias de Oliveira e Silva, Fábio Andrey da Costa Araújo, Marianne de Vasconcelos Carvalho

**Affiliations:** 1 Universidade de Pernambuco, Faculdade de Odontologia de Pernambuco, Programa de Pós-Graduação em Odontologia, Recife, PE, Brasil.; 2 Hospital Universitário Oswaldo Cruz, Centro Integrado de Anatomia Patológica, Recife, PE, Brasil.; 3 Hospital Universitário Oswaldo Cruz, Departamento de Cirurgia e Traumatologia Bucomaxilofacial, Recife, PE, Brasil.

A 36-year-old man presented with a chief complaint of a painful non-healing lesion on the tongue, with a development time of approximately 60 days. Physical examination revealed a poorly defined ulcerative lesion affecting the tongue apex (Figure 1). Lymphadenopathy was not observed. The patient reported previous use of triamcinolone acetonide for over 30 days without any improvement. Hematological examinations were within normal limits, and serological tests were negative for human immunodeficiency virus (HIV), syphilis, and hepatitis. An incisional biopsy was performed to assist with the diagnosis. Microscopically, the lesion showed granulomatous inflammation, composed of multinucleated giant cells, epithelioid histiocytes, and lymphocytes (Figure 2A). Ziehl-Neelsen staining was positive for acid-fast bacilli (Figure 2B), leading to the diagnosis of tuberculosis. Neither chest imaging alterations nor other signals of pulmonary or systemic involvement were observed. Sixty days after starting antituberculous therapy, the patient presented with complete healing of the oral lesion (Figure 3). After six months, no signs of relapse were observed.

**FIGURE 1:**
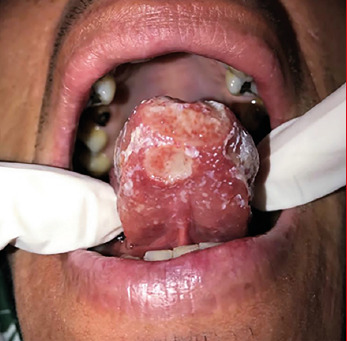
Initial clinical presentation of the ulcerative lesion affecting the apex of the tongue.

**FIGURE 2:**
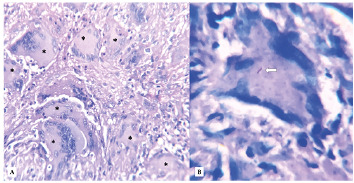
**(A)** Photomicrography showing multiple multinucleated giant cells (*), arranged along with inflammatory infiltrate composed primarily of epithelioid histiocytes and lymphocytes (Hematoxylin and eosin; original magnification ×400). **(B)** Photomicrography showing acid-fast bacillus (arrow) inside a multinucleated giant cell (Ziehl-Neelsen stain; original magnification ×1000).

**FIGURE 3:**
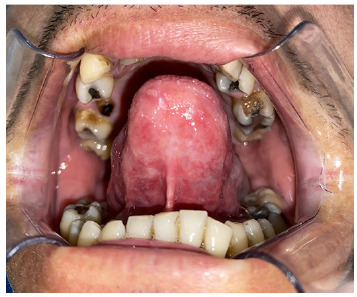
Complete healing of the ulcerative lesion in the tongue after two months of antituberculous therapy.

Primary oral lesions caused by tuberculosis occur without pulmonary infection and are extremely rare, occurring in less than 1% of extrapulmonary cases^1^ ^,^ ^2^. The diagnosis of oral tuberculosis is challenging because the lesions are difficult to differentiate from other ulcerative conditions of the oral mucosa^3^. Despite its rarity, it should be considered in differential diagnosis because tuberculous lesions in the oral cavity may be the only manifestation of primary or secondary disease. Therefore, early diagnosis and proper treatment are fundamental to avoid complications and reduce the spread of infection in the community.

## ETHICS

All procedures performed in studies involving human participants were in accordance with the ethical standards of the Institutional Research Committee as well as the 1964 Helsinki Declaration and its later amendments or other comparable ethical standards.
